# The technique of 3D reconstruction combining with biochemistry to build an equivalent formula of indocyanine green (ICG) clearance test to assess the liver reserve function

**DOI:** 10.1186/s12893-020-00952-z

**Published:** 2020-11-12

**Authors:** Jinli Zheng, Wei Xie, Yang Huang, Yunfeng Zhu, Li Jiang

**Affiliations:** 1 Department of Liver Surgery, Liver Transplantation Center, West China Hospital of Sichuan University, Chengdu, Sichuan China; 2 Department of Radiology Department, West China Hospital of Sichuan University, Chengdu, Sichuan China

**Keywords:** Hepatectomy, Liver reserve function, ICG clearance test, ICG 15 min retention rate

## Abstract

**Background:**

The indocyanine green (ICG) clearance test is the main method of evaluating the liver reserve function before hepatectomy. However, some patients may be allergic to ICG or the equipment of ICG clearance test was lack, leading to be difficult to evaluate liver reserve function. We aim to find an alternative tool to assist the clinicians to evaluate the liver reserve function for those who were allergic to the ICG or lack of equipment before hepatectomy.

**Methods:**

We retrospected 300 patients to investigate the risk factors affecting the liver reserve function and to build an equivalent formula to predict ICG 15 min retention rate (ICG-R15) value.

**Results:**

We found that the independent risk factors affecting ICG clearance test were total bilirubin, albumin, and spleen-to-non-neoplastic liver volume ratio (SNLR). The equivalent formula of the serological index combining with SNLR was: ICG-R15 = 0.36 × TB (umol/L) − 0.78 × ALB(g/L) + 7.783 × SNLR + 0.794 × PT (s) − 0.016 × PLT(/10^9^) − 0.039 × ALT (IU/L) + 0.043 × AST (IU/L) + 23.846. The equivalent formula of serum index was: ICG-R15_2_ = 24.665 + 0.382 × TB (umol/L) − 0.799 × ALB(g/L) − 0.025 × PLT(/10^9^) + 0.048 × AST(IU/L) − 0.045 × ALT(IU/L). And the area under the ROC curve (AUC) of predicting ICG-R15 ≥ 10% was 0.861 and 0.857, respectively.

**Conclusion:**

We found that SNLR was an independent risk factor affecting liver reserve function. Combining with SNLR to evaluate the liver reserve function was better than just basing on serology.

## Background

Hepatectomy is still as the first-line treatment for the patients with hepatic nodules, especially for hepatocellular carcinoma [1–3]. Though the liver transplantation is the optimal treatment for the early stage hepatocellular carcinoma [4], the lack of organs limits its feasibility. With the development of surgical techniques and preoperative managements, the postoperatvie complications have drown from 20% to 3–5% [5–7]. Nowadays, the posthepatectomy liver failure (PHLF) is the main reason of the perioperative death, which is mainly caused by the insufficient residual liver function [8, 9]. Therefore, it is still necessary to evaluate the liver reserve function before hepatectomy.

There are several methods of evaluating the liver function, including Child -Pugh score [10], model for end-stage liver disease (MELD) score [11] and indocyanine green (ICG) test [12]. Child–Pugh score system is the most common methods of evaluating the liver function, which is classified by total bilirubin (TB), albumin (ALB), prothrombin time (PT), ascites and psychosis (hepatic encephalopathy HE). From these criteria to classify the liver function, it would be influenced by assessors’ awareness. And with the increasing application of blood products in the clinic, which have also become an important factor affecting the assessor to judge the situation of liver function. On the other hand, some previous studies have reported that the patients with Child–Pugh A class would have a significantly distinct liver function [13, 14]. MELD score is commonly used to evaluate the patients in waiting list of liver transplantation [15]. ICG test can evaluate the liver reserve function safely and accurately, and this method is widely used in the East [16]. The previous studies showed that when ICG-R15 was no more than 10%, the patients can be tolerant of having a major hepatectomy [17]. However, 0.7% patients would occur adverse reactions when they were injected the ICG from a vein [18]. So, there needed an alternative tool to assist surgeons to evaluate the live reverse function, when the patients occurred the adverse reactions or the equipment of ICG clearance test was lack.

Kawamura et al. has put forward an equivalent formula of ICG-R15 in 2008 [19], which was combined with single-photon emission computer tomography (SPECT) to assess the liver reserve function. However, SPECT needs complicated operating procedures and the cost is expense, so it is difficult to apply in clinic, especially for the developing country. In 2017, Pan-Kin et al. [20] reported that the ICG-R15 value was associated with TB, ALB, PT and PLT (platelet count), meaning that the liver reserve function can be reflected by serology index. On the other hand, the liver-spleen volume ratio can be as a risk factor for predicting the safety of hepatectomy [21]. So we hypothesized that the spleen volume might have a relationship with the liver reserve function and we projected to find an alternative tool to evaluate liver reserve function for the surgeons to estimate the liver reserve function before hepatectomy, combining with spleen volume and serology index.

## Methods

### Patients

We enrolled 300 patients randomly in our center, liver surgery and liver transplantation center, West China hospital, Sichuan University, from 2012 to 2016 January. The criteria as: (1) Age > 18 years; (2) No history of treatment for other tumors, such as colon cancer, gastric cancer, etc.; (3) No fatal underlying diseases, such as heart disease, respiratory insufficiency, etc.; (4) Abdominal CT or MRI related examination was performed in our hospital; (5) The level of serum total bilirubin was twice as high as the normal level (< 60 μmol/L); (6) All patients were received ICG clearance test in our hospital.

### Calculation of SNLR

IQQA LIVER software (EDDA Technology, Princeton, NJ), an automatic 3D organ reconstruction of liver/spleen, was used to perform the volumetric analyzes on liver and spleen, and to measure liver and spleen volume. This software can also measure the tumor volume of liver. The non-neoplastic liver volume (NLV) was calculated as: NLV = Total liver volume − lesion volume. The preoperative spleen-to-non-neoplastic liver volume ratio (SNLR) was calculated as: SNLR = [spleen volume/NLV]. The 3D organ reconstruction was performed by Wei Xie, a 5-year experienced radiologist.

### The procedures of 3D reconstruction

We copied the images of CT or MRI by blank CD-ROM, and then put the images into the IQQA LIVER software by computer, which has been installed in the computer of our center. By the IQQA LIVER software, we could draw the shape of liver, tumor and spleen, event the hepatic vein, potal vein and hepatic artery. Therefore, we reconstructed the shape and calculated the volume of liver, tumor and spleen, respectively. Further more, we could predict the volume of residual liver before hepatectomy, such as the resection of hepatocellular carcinoma, hepatic adenomas and living donor liver transplantation.

### Statistical analysis

All data were analyzed by SPSS22.0. We divided the ICG-R15 into two groups: ≥ 10% and < 10%. The continuous variables were expressed as mean and standard deviation ($\overline{\text{x}} + {\text{sd}}$) or median and interquartile, and the categorical variables were presented as number and percentages. Two sample T test or Wilcoxon sign-rank test were performed to analyze the continuous variables. Chi-square (χ^2^) test or Fisher exact test were used to analyze the categorical variables. The multivariate logistic regression analysis was carried out to identify the independent risk factors affecting the ICG-R15 value, combining with the significant variables in two sample T test, Wilcoxon sign-rank test, Chi-square test or Fisher exact test. The receiver operating characteristic (ROC) was used to predict diagnostic efficacy and to confirm the cut-off values of the independent risk factors. All independent risk factors were taken into propensity score match (PSM), except for SNLR, to identify the SNLR could reflect the liver reserve function, independently.

The multiple linear regression analysis of the ICG-R15 value was carried out to obtain the linear relation of the ICG-R15 value in three situations (the linear relation combining with SNLR before PSM, the linear relation combining with SNLR after PSM and the linear relation based on serology). The paired T test or paired Wilcoxon sign-rank test was used to analyze the difference between actual ICG-R15 (aICG-R15) value and estimated ICG-R15 (eICG-R15) value in three situations. ROC was used to assess the ability of eICG-R15 predicting aICG-R15 ≥ 10%. All the tests were statistically significant with *p* < 0.05.

## Results

### The baseline of the patients before and after PSM

As showing in the Table 1, there were 97 patients with ICG-R15 ≥ 10% and 203 patients with ICG-R15 < 10%. The age, TB, ALT, AST, spleen volume and SNLR were significantly higher in ICG-R15 ≥ 10% patients than ICG-R15 < 10% patients, and the difference was significant. On the other hand, the HGB, WBC, PLT and ALB were lower than patients with ICG-R15 < 10%, and the difference was significant. The other situations, such as sex, BMI, HBV, the tumor volume and the non-neoplastic liver volume were not significant.

**Table 1:** Baseline characteristic of patients with ICG-R15 ≥ 10% or ICG-R15 < 10%

Before propensity matching				After propensity matching		
	≥ 10% (n = 97)	< 10% (n = 203)	*p-*value	≥ 10% (n = 58)	< 10% (n = 58)	*p-*value
Age (Y)	55.57 ± 11.12	51.68 ± 12.24	0.009*	55.07 ± 11.65	54.09 ± 12.03	0.656
Sex (male, %)	72 (74.23%)	167 (82.27%)	0.106	45 (77.59%)	52 (89.66%)	0.079
BMI	23.39 ± 3.24	22.93 ± 3.07	0.232	23.53 ± 3.31	22.55 ± 3.39	0.117
HGB (g/L)	128.52 ± 25.66	138.49 ± 23.92	0.001*	131.76 ± 28.53	134.57 ± 25.56	0.578
WBC(× 10^9^ /L)	4.96 ± 2.54	5.72 ± 1.86	0.005*	5.37 ± 2.81	5.41 ± 2.03	0.928
PLT (× 10^9^ /L)	112.62 ± 72.50	153.88 ± 76.22	< 0.001*	122.19 ± 60.93	138.29 ± 77.14	0.215
HBV (positive, %)	77 (79.38%)	145 (71.43%)	0.241	48 (82.76%)	41 (70.69%)	0.125
Tb (μmol/L)	23.58 ± 12.90	15.88 ± 7.81	< 0.001*	18.80 ± 9.44	19.47 ± 10.32	0.716
AST (median IU/L)	56.0 (39.0–74.0)	42.0 (27.0–62.0)	< 0.001*	49.00 (37.75–71.75)	52.50 (32.50–71.05)	0.722
ALT (median IU/L)	45.0 (27.0–71.0)	39.0 (22.0–60.0)	0.030*	41.50 (27.00–55.25)	44.50 (21.75–84.25)	0.647
ALB (g/L)	36.8 ± 4.85	41.12 ± 4.38	< 0.001*	38.25 ± 4.61	38.15 ± 3.99	0.897
PT(s)	12.28 ± 1.12	12.59 ± 1.49	0.071	12.64 ± 1.40	12.57 ± 1.11	0.753
TV (mL)	78.9 (22.49–386.29)	130.(45.99–405.99)	0.137	88.86 (25.25–451.53)	156 (81.04–611.76)	0.107
RLV (mL)	1152.65 ± 358.80	1121.30 ± 268.05	0.398	1128.72 ± 292.35	1122.33 ± 326.21	0.912
SV (mL)	471.57 ± 282.31	284.12 ± 180.39	< 0.001*	414.41 ± 210.77	324.82 ± 206.34	0.023*
BSA	1.66 (1.55–1.75)	1.65 (1.53–1.77)	0.797	1.67 (1.58–1.76)	1.63 (1.52–1.77)	0.344
SNLV	703.85 ± 215.92	673.60 ± 140.22	0.147	689.79 ± 167.03	676.81 ± 166.46	0.805
SNLR	0.44 ± 0.29	0.26 ± 0.16	< 0.001*	0.38 ± 0.22	0.30 ± 0.18	0.029*

^*^ Reflecting the difference was significant in statistics (p < 0.05)

We took the significant variables into PSM, except for spleen volume and SNLR. The allowable error of selection was a = 0.1. After PSM, 58 pair patients were obtained. And we found that the difference of age, TB, AST, ALT, HGB, WBC, PLT and ALB were not significant after PSM, just only the spleen volume (414.41 ± 210.77 vs 324.82 ± 206.34, p = 0.023) and the SNLR (0.38 ± 0.22 VS 0.30 ± 0.18, p = 0.029) were significant, indicating that the PSM results were credible.

### The results of logistic regression analysis

Table 2 was the result of logistic regression analysis. We found that the TB, ALB, HBV, age, SNLR were the risk factors of ICG-R15 value before PSM. As to the SNLR index including spleen volume and non-neoplastic liver volume, we did not include these two values in the logistic regression analysis. Combining with Table 1, we can find that TB, ALB, age, SNLR were the independent risk factors for ICG-R15 value. The logistic regression analysis after PSM showed that BMI and SNLR were the factors affecting the value of ICG-R15, but SNLR was the independent factor for ICG-R15, indicating that the PSM has eliminated the mixed factors.

**Table 2:** The result of logistic regression analysis

Variables	β	SE	Wald *χ*^2^	RR	IC (95%)	*p-*value
The logistic regression analysis of ICG-R15 before PSM						
TB	0.093	0.018	27.351	1.098	(1.060, 1.137)	< 0.001
ALB	0.238	0.041	33.722	1.268	(1.171, 1.374)	< 0.001
HBV	0.991	0.448	4.885	2.694	(1.119, 6.489)	0.027
SNLR	3.088	0.932	10.986	21.943	(3.533, 136.274)	0.001
Age	0.056	0.016	12.441	1.058	(1.025, 1.092)	< 0.001
The logistic regression of analysis ICG-R15 after PSM						
BMI	0.120	0.060	3.986	1.127	(1.002, 1.268)	0.046
SNLR	2.552	1.059	5.804	12.827	(1.609, 102.230)	0.016

### The diagnostic efficiency of the independent factors

**Fig. 1:**
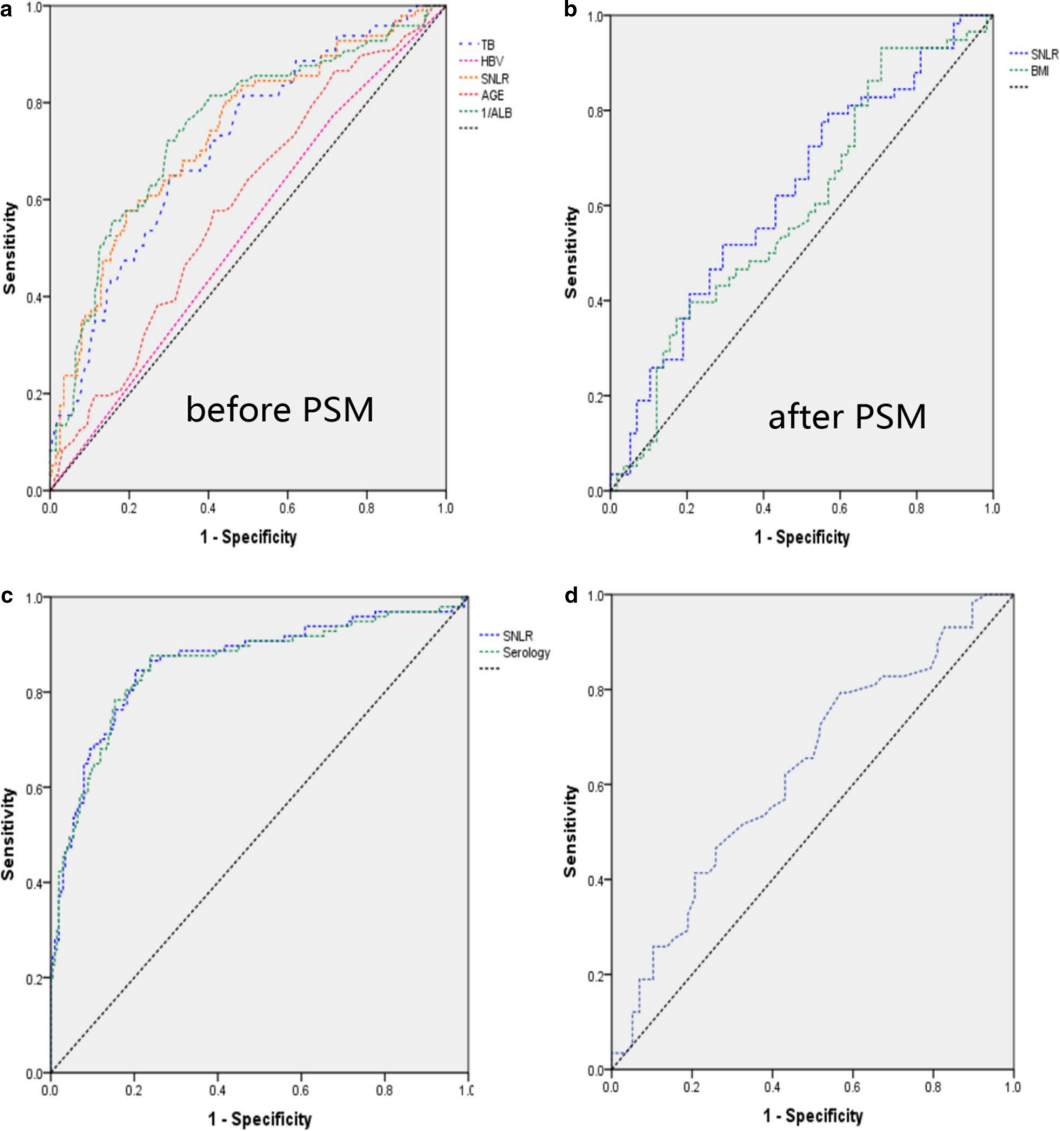
**a** The ROC curves of variables in predicting ICG-R15 ≥ 10% before PSM, and the factors were as: TB, 1/ALB, age, SNLR and HBV. The AUC was 0.712, 0.747, 0.589, 0.733, 0.529, respectively, and the best cut-off point was 17.5 μmol/L, 0.0256, 55.5 years old, 0.3394, respectively. **b** The ROC curves of variables in predicting ICG-R15 ≥ 10% after PSM, and the factors were as: SNLR and BMI. The best cut-off point was 0.2332 and 20.0. **c** The ROC curves of the eICG-R15 calculated by the formulas (ICG-R15 and ICG-R15_2_) respectively, to predict the actual ICG-R15 ≥ 10%, and the AUC was 0.861 and 0.857 respectively. **d** The ROC curves of the eICG-R15 calculated by the formula (ICG-R15_1_) to predict the actual ICG-R15 ≥ 10%, and the AUC was 0.628

Table 3 showed the diagnostic efficacy of independent risk factors before and after PSM in predicting ICG-R15 ≥ 10%. The area under the ROC curve (AUC) of TB, 1/ALB (the ALB as a protect factor for the liver reserve function, if we used the direct serum ALB to determine the optimal concentration of serum ALB, the AUC would be less than 0.5, so we adopted the reciprocal of serum ALB (1/ALB) to determine the optimal concentration of serum ALB), age and SNLR were 0.712, 0.747, 0.589 and 0.733, respectively (Fig. 1a). And the best cut-off values were 17.45 μmol/L, 0.0256 (ALB was 39.06 g/L), 55.5 years old and 0.3397 (Table 3). After PSM, the SNLR was the independent risk factor for the ICG-R15, and the AUC was 0.626 (Fig. 1b).

**Table 3:** Diagnostic efficacy of ICG-R15 ≥ 10% before and after PSM

	Before PSM					After PSM	
	TB	HBV	SNLR	1/ALB	AGE	BIM	SNLR
AUC	0.712	0.529	0.733	0.747	0.589	0.594	0.626
Yonden index (%)	33.9	5.90	38.5	42.6	0.163	22.4	22.4
Sensitivity (%)	64.9	77.3	57.7	72.2	57.7	93.1	79.3
Specificity (%)	69.0	28.6	80.8	70.4	58.6	29.3	43.1
Best cut-off	17.45	-	0.3397	0.0256	55.5	20.0	0.2332

### The results of multiple linear regression analysis

**Fig. 2:**
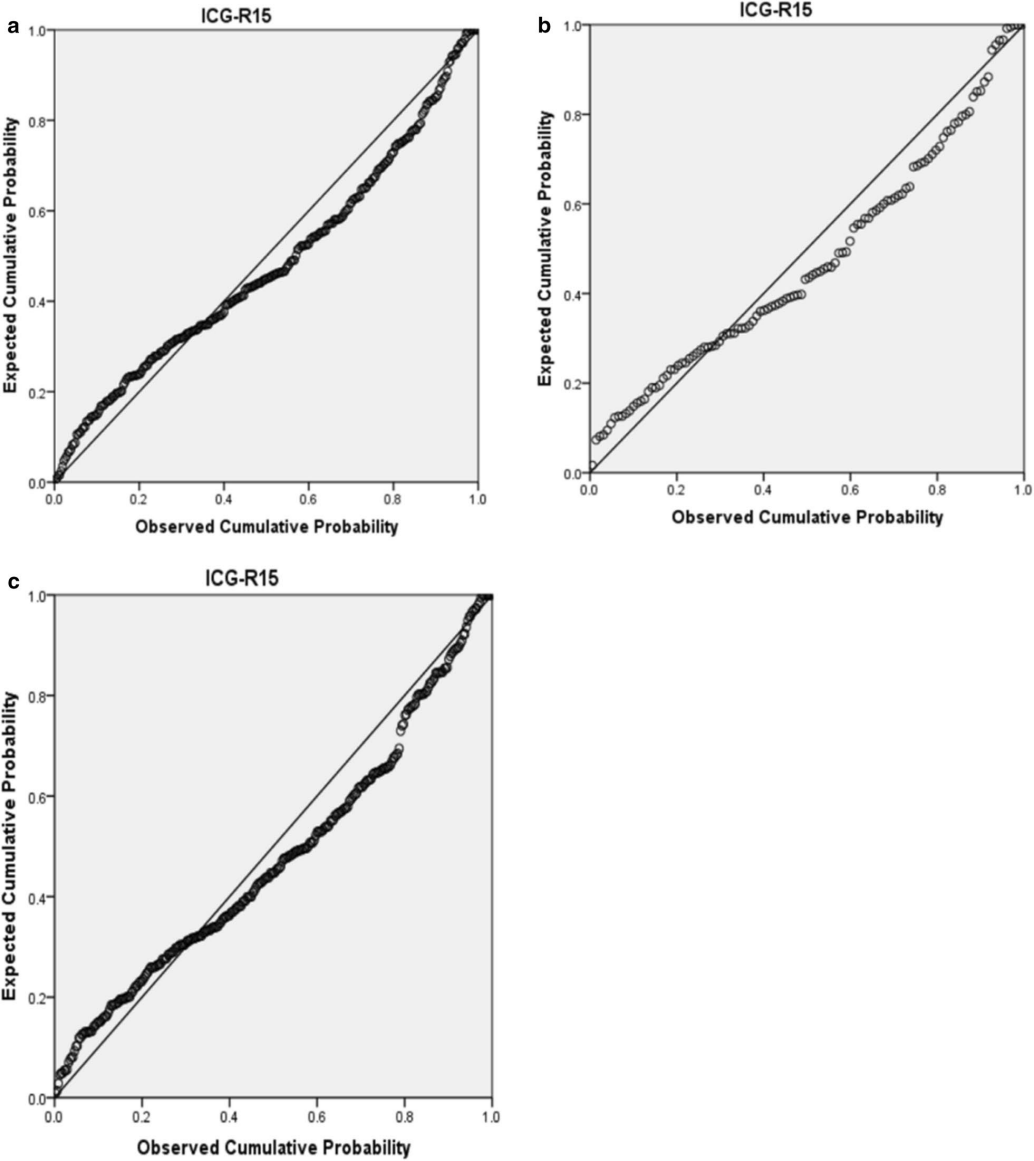
**a** The P-P diagram of the expected cumulative probability and observed cumulative probability of ICG-R15 value before PSM. **b** The P-P diagram of the expected cumulative probability and observed cumulative probability of ICG-R15 value after PSM. **c** The P-P diagram of the expected cumulative probability and observed cumulative probability of ICG-R15 value just based on serological index

Table 4 showed the results of multiple regression analysis of ICG-R15 value, before and after PSM. Before PSM combining with SNLR, the expression formula was as following: ICG-R15 = 0.36 × TB (μmol/L) − 0.78 × ALB(g/L) + 7.783 × SNLR + 0.794 × PT(s) − 0.016 × PLT (/10^9^) − 0.039 × ALT (IU/L) + 0.043 × AST(IU/L) + 23.846 (R^2^ = 0.507), and the linear distribution result was shown in Fig. 2a. After PSM combining with SNLR, the formula was as: ICG-R15_1_ = 15.638 × SNLR + 6.734 (R^2^ = 0.119), and the linear distribution was shown in Fig. 2b. The linear regression analysis on patients just based on serological indicators to obtain the relevant serological equivalent formula of ICG-R15 value: ICG-R15_2_ = 24.665 + 0.382 × TB (umol/L) − 0.799 × ALB(g/L) − 0.025 × PLT (/10^9^) − 0.048 × AST(IU/L) − 0.045 × ALT (IU/L), and the linear distribution result was shown in Fig. 2c.

**Table 4:** The results of multiple linear regression analysis

Variables	β	SE	T	*p-*value	*R*^2^
The relationship of ICG-R15 combined with SNLR before PSM					
TB	0.360	0.046	7.769	< 0.001	0.507
ALB	− 0.780	0.093	− 8.361	< 0.001	
SNLR	7.783	2.270	3.429	0.001	
PT	0.794	0.356	2.235	0.026	
PLT	− 0.016	0.006	− 2.535	0.012	
ALT	− 0.039	0.011	− 3.508	0.001	
AST	0.043	0.013	3.222	0.001	
Constant	23.846	6.723	3.547	< 0.001	
The relationship of ICG-R15 combined with SNLR after PSM					
SNLR	15.638	1.622	4.152	< 0.001	0.119
Constant	6.734	2.058	3.853	< 0.001	
The relationship of ICG-R15 based on serology					
TB	0.382	0.047	8.202	< 0.001	0.487
ALB	− 0.799	0.095	− 8.435	< 0.001	
PLT	− 0.025	0.006	− 4.385	< 0.001	
PT	1.058	0.353	2.004	0.003	
ALT	− 0.045	0.011	-4.016	< 0.001	
AST	0.048	0.013	3.624	< 0.001	
Constant	24.665	6.841	3.605	< 0.001

All formulas were calculated for the ICG-R15 values, called estimated ICG-R15 values (eICG-R15). Paired T-test, paired rank sum (W) test and chi-square (χ^2^) test were performed to compare the difference with actual ICG-R15 (aICG-R15), respectively, as showing in Table 5. There was no significant difference between the aICG-R15 and eICG-R15 values before PSM combining with SNLR (10.04 ± 10.04 vs 10.05 ± 7.45, *p* = 0.984). And the W-test suggested that the distribution of aICG-R15 and eICG-R15 was no significant difference (6.05(3.43–12.95) vs 8.64 (5.12–13.69), *p* = 0.092). The diagnostic efficiency of ICG-R15 ≥ 10% was as following: sensitivity = 84.5%, specificity = 79.7% and AUC = 0.861 (Table 6, Fig. 1c). The difference between eICG-R15_1_ and aICG-R15 after PSM combining with SNLR was no significance (12.11 ± 2.35 vs 1 2.10 ± 3.23, *p* = 0.996 and 9.95 (5.13–15.08) vs 11.35 (9.90–13.15), *p* = 0.189, Table 5). However, the AUC was 0.628 and the sensitivity was 79.3%, specificity was 43.1% (Table 6, Fig. 1d). The difference between eICG-R15_2_, based on serological index, and actual ICG-R15 was no significance (10.04 ± 10.04 vs 10.11 ± 7.29, *p* = 0.877 and 6.05(3.43–12.95) vs 9.26(5.06–13.87), *p* = 0.060), and the AUC was 0.857, sensitivity was 87.6%, specificity was 76.2% (Table 6, Fig. 1e).

**Table 5:** The comparison between estimated-value and actual-value

	Actual value	Estimated value	T/W/χ^2^	*p-*value
ICG-R15 combined with SNLR (before PSM)				
ICG-R15 (%)	10.04 ± 10.04	10.05 ± 7.45	− 0.20	0.984
ICG-R15 (%)	6.05 (3.43–12.95)	8.64 (5.12–13.69)	24,943.000	0.092
ICG-R15 ≥ 10% (n)	97	127	6.411	0.011
ICG-R15 combined with SNLR (after PSM)				
ICG-R15 (%)	12.11 ± 2.35	12.10 ± 3.23	− 0.005	0.996
ICG-R15 (%)	9.95(5.13–15.08)	11.35 (9.90–13.15)	3870.000	0.189
ICG-R15 ≥ 10% (n)	58	87	15.467	< 0.001
ICG-R15 purely based on serology				
ICG-R15 (%)	10.04 ± 10.04	10.11 ± 7.29	− 0.142	0.877
ICG-R15 (%)	6.05(3.43–12.95)	9.26 (5.06–13.87)	25,237.000	0.060
ICG-R15 ≥ 10% (n)	97	132	8.651	0.003

**Table 6:** Diagnostic efficacy of estimated-value predicting actual-value

	AUC	Yonden index (%)	Sensitivity (%)	Specificity (%)	Best cut-off point
Combined with SNLR to predict ICG-R15 ≥ 10% (Before PSM)	0.861	0.643	84.5	79.7	10.24
Combined with SNLR to predict ICG-R15 ≥ 10% (After PSM)	0.628	0.224	79.3	43.1	10.41
Purely serology to predict ICG-R15 ≥ 10%	0.857	0.638	87.6	76.2	10.12

## Discussion

The study was mainly focused on 3D reconstruction technique to evaluate liver reserve function, and combined with clinical serological index to draw a formula to assist the surgeon to assess the liver reserve function. The ICG clearance test can assess the liver reserve function safely and accurately [17], therefore, we took the ICG-R15 value as a reference index for liver reserve function. From the Table 1, the liver reserve function was influenced by many factors, such as age, HGB, PLT, ALB, TB, ALT, AST and SNLR. Among these affected factors, TB, ALB, age, SNLR were independent risk factors of ICG-R15 value. TB affected the ICG-R15 value, mainly related to the metabolism of bilirubin. When the apoptosis of red blood cell, HGB was released into the blood and transported to the liver combining with serum protein to be ingested by hepatocytes and converted into bilirubin, excreted through the biliary tract. On the other hand, ICG was transported to the liver by serum albumin, and it was excreted by the prototype via the biliary tract. Therefore, bilirubin has a competitive, inhibitory relationship with ICG. Meaning that when the TB is increasing, it may inhibit the speed of ICG transported to the liver and affect the ICG-R15 value [22]. Especially for the patient with biliary obstruction, the accuracy of the ICG clearance test was significantly affected [23]. Though the study had excluded patients with TB higher than 2 times of the normal, the influence of TB didn’t completely eliminate. And we found that when TB was higher than 17.45 μmol/L, it would have a greater impact on the ICG clearance test. In additional, ALB as a transporter of the ICG [24, 25], when serum albumin decreased, it would affect the clearance rate of ICG. In the study, we found that when the serum albumin was lower than 39.6 g/L, it might affect the clearance rate of ICG. Age as an independent risk factor affecting the ICG clearance test, it would be mainly related to chronic hepatitis B virus (CHB). Although there was no significant difference of the patients with HBV between the groups (79.38% vs 71.43%, p = 0.241), the age was higher in ICG-R15 ≥ 10% group patients than the ICG-R15 < 10% group patients (55.57 ± 11.12 vs 51.68 ± 12.24, p vs 71.43%, *p* = 0.009). Previous studies have shown that patients with HBV would make progress to cirrhosis or even decompensation [26, 27]. Furthermore, HBV can be as chronic infection, and the carriers might be with a normal liver function, which didn’t cause their attention, leading it difficult to determine the time of being infected by HBV. The patients with ICG-R15 ≥ 10% would be infected for a longer time than the patients with ICG-R15 < 10%, which resulted into more severe cirrhosis than the patients with ICG-R15 < 10%, therefore, the time of being infected by the HBV was a risk factor of ICG-R15 value. However, we can’t identify when the patients were infected by the HBV, and the age might reflect the time of the patients who were infected by HBV, indirectly. So we could take the age as a reference of the time of being infected by HBV when we evaluated the liver reserve function, especially for the patients with an age older than 55.5 years old.

The volume of spleen in patients with ICG-R15 ≥ 10% was larger than the patients with ICG-R15 < 10% (471.57 ± 282.31 vs 284.12 ± 180.39, *p* < 0.001, Table 1), and SNLR was also higher (0.44 ± 0.29 vs 0.26 ± 0.16, p < 0.001). The spleen volume is mainly related to cirrhosis. As the cirrhosis increasing, the pressure of hepatic sinus would increase, showing the intrahepatic pressure increasing, resulting in the portal vein pressure increasing. The portal vein pressure which has increased was an obstacle for the splenic vein, resulting the increasing spleen volume and hypersplenism. The hypersplenism would destruct the PLT, so the PLT was lower in ICG-R15 ≥ 10% group (112.62 ± 72.50 vs 153.88 ± 76.22, p < 0.001, Table 1). By logistic regression analysis, we found that SNLR was an independent risk factor of the ICG-R15 value, and when SNLR ≥ 0.3397, meaning that the patients were 21.943 times to have the ICG-R15 ≥ 10% than those who not. After PSM, eliminating other affected factors of the ICG-R15 value, the SNLR was still higher in ICGR 15 ≥ 10% group (0.38 ± 0.22 vs 0.30 ± 0.18, p = 0.029, Table 1), indicating the PSM was reliable. SNLR was the independent risk factor of ICG-R15 value, but it was unreliable to predict the ICG-R15 ≥ 10% if just only considering the affection of SNLR when we evaluated the liver function (the AUC was 0.626, which was smaller than the AUC combining with serological indicators (the AUC = 0.733)). Therefore, estimating the ICG-R15 value should combine with other risk factors.

From multiple linear regression analysis, we got the SNLR-related formula for the ICG-R15 value [ICG-R15 = 0.36 × TB (μmol/L) − 0.78 × ALB(g/L) + 7.783 × SNLR + 0.794 × PT(s) − 0.016 × PLT(/10^9^) − 0.039 × ALT (IU/L) + 0.043 × AST(IU/L) + 23.846]. The difference of eICG-R15 and aICG-R15 was no significance, indicating that the eICG-R15 value was reliable. Additionally, the coefficient of SNLR was the largest in the formula, indicating that SNLR had the greatest affecting on liver reserve function. The level of AST and ALT can be used as the related variable, mainly because the two enzymes were higher in liver cells and they would be released into the blood when the liver cells were damaged or died, reflecting the liver function situation. On the other hand, from the formula of purely serological index, the difference of eICG-R15 and aICG-R15 was also no significance, indicating that the formula of combining with SNLR and the formula of basing on serological index were comparable.

Furthermore, few studies had reported that the spleen can promote cirrhosis. This was related to the GFT-β1, which could activate the stellate cells, increasing extracellular matrix synthetizing and inhibiting the synthesis of collagenase and matrix metalloproteinase, reducing the decomposition of extracellular matrix, resulting the interstitial deposition in hepatocytes, producing liver fibrosis. However, macrophages in the red pulp of the spleen can secrete GFT-β1, via the portal vein into the liver and participating the process of liver fibrosis. In the cirrhosis model of rat, when the spleen was resected, the GFT-β1 would decrease. It was an evidence for the spleen can synthesise the GFT-β1 [28, 29]. On the other hand, splenectomy can be used as a supportive treatment for the patients with cirrhosis, waiting for liver transplantation, because the splenectomy can slow down the progression of cirrhosis and improve the liver function [30, 31]. Therefore, it was credible to evaluate liver reserve function by SNLR. In this study, We found that the volume of spleen and SNLR were higher in ICG-R15 ≥ 10% patients. The SNLR could reflect the size of spleen volume and non-tumor liver volume in a way. The SNLR was larger meaning the non-tumor liver volume may be smaller, and the liver function was the sum of all normal liver cells function, therefore, SNLR was larger in the patients with ICG-R15 ≥ 10%. Both formulas of basing on serological indicators and combining with SNLR have had no significant difference in predicting actual ICG-R15 values. However, the SNLR could reflect the volume of spleen and liver, and we should choose the method of combining with SNLR to evaluate the liver reserve function.

The volume of spleen played an important role in the recovery of patients after hepatectomy and knowing the status of SNLR may be beneficial for us to choose the surgical methods in pre-operation. Posthepatectomy liver failure (PHLF) was still the main reason of death in patients after hepatectomy, and its incidence was about 7% [32, 33]. The residual liver volume after hepatectomy can be used as the main index to predict PHLF [34]. And there were also some studies suggested that the volume of spleen could affect the recovery of patients. When spleen volume/residual liver volume was higher, the recovery of liver function was slower [35]. After hepatectomy, splenic vein and portal vein blood flow could be increased, promoting the regeneration of hepatocytes [36], so splenectomy for some patients could relieve the progression of liver cirrhosis and the liver function would be better [37, 38]. At the same time, the overload portal venous reflux could lead to damage of liver endothelial cells, inhibit hepatocyte regeneration, and even occur PHLF [39], therefore, the volume of spleen could affect the recovery of patients after hepatectomy. Earlier studies focused on the effect of residual liver volume and spleen volume on postoperative [34], while this study mainly explored the relationship between SNLR and liver reserve function before hepatectomy, and obtained an alternative formula to provide a reference for evaluating the feasibility of surgery. However, the SNLR could reflect the preoperative non-tumor liver volume and spleen volume, indirectly, which can predict the ratio of spleen volume-to-postoperative residual liver volume. Thus, it can provide a reference for the treatment of hepatectomy combined with splenectomy to reduce the incidence of PHLF. On the other hand, the intraoperative bleeding volume, intraoperative blood transfusion volume and intraoperative blocking of portal vein blood flow time can also affect the PHLF [40]. The SNLR can provide reference for the surgeon to choose the methods of operation, but it couldn’t avoid the effect of intraoperative factors (intraoperative bleeding, blood transfusion, portal vein blocking time, etc.) on the PHLF. Therefore, it was still necessary to control the intraoperative bleeding. Furthermore, the indication of splenectomy was mainly based on the size of spleen and the condition of blood cells of the patients. So there needs a large number of clinical randomized controlled trials for SNLR to guide the hepatectomy combined with splenectomy. Furthermore, Siyuan Yao et al. suggested that the spleen volume/graft volume ratio was higher than 0.7, the small-for-size syndrome (SFSS) was at greater risk after living liver transplantation [26]. Therefore, for recipients and donors undergoing living liver transplantation, the recipient spleen volume and donor available for resection of the liver can be calculated by three-dimensional organ reconstruction technique before operation. Through this way, we can predict the ratio of spleen volume/graft volume before living liver transplantation to provide a reference for the surgeon to consider whether the recipient should undergo splenectomy. On the other hand, the ICG-R15 equivalent formula obtained by serological index, simply, could be used to predict the ICG-R15 value, but it couldn’t provide the volume of spleen and liver. The equivalent combined with SNLR could show the volume of spleen and liver directly, providing a reference for the surgeon before hepatectomy or living liver transplantation. Thus, evaluating the liver reserve function combining with SNLR is better than just based on serological index for evaluating the liver reserve function.

Compared with the previous formulas [19, 20], the formula combined with SNLR could provide the location of tumor and the volume of residual liver, directly, and the eICG value was comparable and the technique of 3D reconstruction was mature. On the another hand, there were other methods to assess the liver reserve funtion, such as LiMAx (liver maximum capacity test, Humedics, Berlin, Germany), 99mTc-sulfur colloid scintigraphy and ultrasound elastography [41–43]. The LiMAx was based on the metabolic function capacity of the cytochrome P450 isoenzyme 1A2 (CYP450 1A2) and could be used to evaluate the liver function, however, we couldn’t know the shape and volume of liver, which could provide a direct evidence for surgeon to choose the method of treatment. The 99mTc-sulfur colloid scintigraphy needed the SPECT to assist the evaluation, and the cost of SPECT was expensive [19]. Ultrasound elastography, measured the velocity of an elastic shear wave propagating of the liver, could detect the early liver fibrosis. The transient elastography (TE), a noninvasive technique of ultrasound elastography to detect the liver fibrosis, has been applied to assess the liver fibrosis for its sensitivity, specificity and reproducibility [44]. However, TE was influenced by ascites and obesity, and it couldn’t provide a directly view for the surgeon to identify the location of tumor and to measure the volume of liver and spleen, which could provide a reference to make a decision before hepatectomy. Compare to the ICG clearance test and combining with the 3D reconstruction technique to assess the liver function, the cost of TE was equal, about 60$ in our center (except for the cost of imaging). As the previous studies reported, the Child-Pugh A class would have a significantly distinct liver function, so it might have a TE test to identify the fibrosis if there was lack the equipment of ICG clearance test and 3D reconstruction.

The limitations of this study were as following: (1) The retrospective study has its owe shortcomings, for example, we can not identify when the patients were infected by HBV, so the age was as an independent risk factor in this study. Actually, the time of being infected by HBV should be as the independent risk factor. (2) There needed a large number of clinical samples for further identifying the relationship of SNLR and ICG-R15, however, as far as we know, this study was the first research combining radiology to evaluate the liver function, which met the trend to combine with many ways to evaluate the liver function. (3) We can’t eliminate the effects of intrahepatic vascular (hepatic artery, hepatic vein, bile duct, etc.) when we reconstructed the 3D model of liver and spleen.

## Conclusion

SNLR was an independent factor for liver reserve function. The equivalent formula of serological index combined with SNLR and the equivalent formula of purely serological index could be used to predict the aICG-R15 value, but the formula of serological index combined with SNLR was better than the formula based on purely serological index.

## Data Availability

The data sets used during the current study are available from the corresponding author on reasonable request.

## Abbreviations

BMI: Body mass index
HGB: Hemoglobin
WBC: White blood cell
PLT: Platelet
HBV: Hepatic B virus
TB: Total bilirubin
ALT: Alanine aminotransferase
AST: Aspartate aminotransferase
ALB: Albumin
PT: Prothrombin time
TV: Tumor volume
NNLV: Non-neoplastic liver volume
SV: Spleen volume
SNLR: Spleen-non-neoplastic liver volume ratio
